# Biofilms in modern CaCO_3_-supersaturated freshwater environments reveal viral proxies

**DOI:** 10.1038/s41598-024-75998-7

**Published:** 2024-10-29

**Authors:** Mirosław Słowakiewicz, Andrzej Borkowski, Edoardo Perri, Paweł Działak, Ezher Tagliasacchi, Michał Gradziński, Sándor Kele, Lars Reuning, Tom Kibblewhite, Fiona Whitaker, R. Pamela Reid, Maurice E. Tucker

**Affiliations:** 1 Faculty of Geology, University of Warsaw, Warsaw, Poland; 2 Faculty of Geology, Geophysics and Environmental Protection, AGH University of Krakow, Kraków, Poland; 3 Dipartimento di Biologia Ecologia e Scienze della Terra, Università della Calabria, Rende, Italy; 4 Faculty of Engineering, Pamukkale University, Kınıklı Campus, Denizli, Turkey; 5 Institute of Geological Sciences, Jagiellonian University, Kraków, Poland; 6 HUN-REN Research Centre for Astronomy and Earth Sciences, Institute for Geological and Geochemical Research, Budapest, Hungary; 7 CSFK, MTA Centre of Excellence, Budapest, Hungary; 8 Institute of Geosciences, Kiel University, Kiel, Germany; 9 School of Earth Sciences, University of Bristol, Bristol, UK; 10 Rosenstiel School of Marine, Atmospheric and Earth Science, University of Miami, Miami, USA

**Keywords:** Biogeochemistry, Environmental sciences

## Abstract

Biofilms are mucilaginous-organic layers produced by microbial activity including viruses. Growing biofilms form microbial mats which enhance sediment stability by binding particles with extracellular polymeric substances and promoting growth through nutrient cycling and organic matter accumulation. They preferentially develop at the sediment-water interface of both marine and non-marine environments, and upon the growing surfaces of modern tufa and travertine. In this context, however, little is known about the factors, environmental or anthropogenic, which affect viral communities in freshwater spring settings. To explore this issue, geochemical and metagenomic data were subjected to multidimensional analyses (Principal Component Analysis, Classical Multidimensional Scaling, Partial Least Squares analysis and cluster analysis based on beta-diversity), and these show that viral composition is specific and dependent on environment. Indeed, waters precipitating tufa and travertine do vary in their geochemistry with their viruses showing distinct variability between sites. These differences between virus groups allow the formulation of a viral proxy, based on the Caudoviricetes/Megaviricetes ratio established on the most abundant groups of viruses. This ratio may be potentially used in analysing ancient DNA preserved in carbonate formations as an additional source of information on the microbiological community during sedimentation.

**Supplementary Information:**

The online version contains supplementary material available at 10.1038/s41598-024-75998-7.

## Introduction

Biofilms, which are chiefly formed by algae, bacteria, fungi, viruses, protozoans and metazoans, commonly result in adhesion of bacterial cells to organic and inorganic surfaces. After adhesion, microbial cellular aggregates that are enclosed in a mucilage of extracellular polymeric substances (EPS;^1^) living under a wide range of conditions and environments, from freshwater to hypersaline ecosystems^2,3^, where they play a key role in the precipitation of carbonates and other minerals. In freshwater biofilms mineral precipitation is mostly mediated by microbial EPS^4–11^ but also through the activity of viruses^12^, eukaryotes and fungi^13^. Good examples of modern terrestrial environments are springs where the main products of microbial and viral activity are carbonates precipitated from CaCO_3_-supersaturated waters producing tufa and travertine. In these carbonate-rich systems, conditions which favour processes leading to precipitation of minerals are temperature (> 30 °C in travertine and < 20 °C in tufa), HCO_3_^−^ content (> 6 in travertine and < 6 mmol/L in tufa), pH (6.5–7.9 in travertine and 8-8.5 in tufa) and flow rate (150–500 in travertine and 2–80 L/s in tufa^14–17,11^) including tectonics and seasonal climatic variations^18^. Here, biofilms and their microbial components are the main agents of mineralisation, typically leading to laminated or clotted carbonate deposits^11,12^. As has been recently shown from travertines occurring around Europe and Asia Minor, viruses turn out to have significantly influenced organomineralisation in biofilms^12^. However, the concentration of viruses and their variation with reference to pH, temperature, water composition and climatic zones on a more global scale, have not been examined so far in modern terrestrial systems where carbonate precipitation is taking place. Most information about viral activity in freshwater ecosystems comes from analyses of water and bottom sediments in lakes and rivers, e.g.^19,20^, or microbialites^21,22^ and one site of a calcareous tufa^23^. Carbonate precipitation in microbialites is mainly driven by the interaction of microbial growth and metabolism with mineral precipitation and grain trapping^24,25^. In the case of tufa and travertine deposits, mineral precipitation results from the oversaturation of the waters in association with springs with respect to CaCO_3_ in conjunction with microbial growth. Marine systems in which viruses occur and become significant contributors to the total biomass developing microbial mats and microbialites (i.e., stromatolites, thrombolites) are better documented^26–31^. In this context, viruses also occur in extreme environments including hydrothermal vents^32^, acid mine drainages^33^ and deep subsurface rocks^34,35^.

A crucial current issue pertains to the revision of virus taxonomy^36^, which has led to extensive changes in nomenclature from one based on morphology to a system based on phylogenetic relationships. This has resulted in the establishment of virus groups often with indeterminate systematic positions at various taxonomic levels. Additionally, new approaches to analysing viral communities in diverse environments, especially in the highly specific environment of carbonate mineralisation, must consider the aforementioned challenges. It remains unclear as to which taxonomic level should be adopted as the most representative for characterising the analysed environments. Therefore, it seems that analyses should address as wide a range of taxonomic levels as possible. First, the new taxonomy is not comparable to that used in earlier work characterising viruses in the environment. Second, the lowest taxonomic levels (*Genus* and *Species* levels) may have less diversity due to the lack of identification of many viral sequences at these levels. The presented study represents the first attempt to characterise contemporary carbonate sedimentary environments in light of the recently proposed virus taxonomy. From this, it can be hypothesised that travertine and tufa environments have differentiated viral compositions influenced either by environmental chemical factors and/or temperature, and this approach has allowed us to establish virus-based proxies for both carbonate systems. This new proxy seems particularly important for viruses entrapped in biofilms.

## Results

### Geochemical analyses

**Fig. 1:**
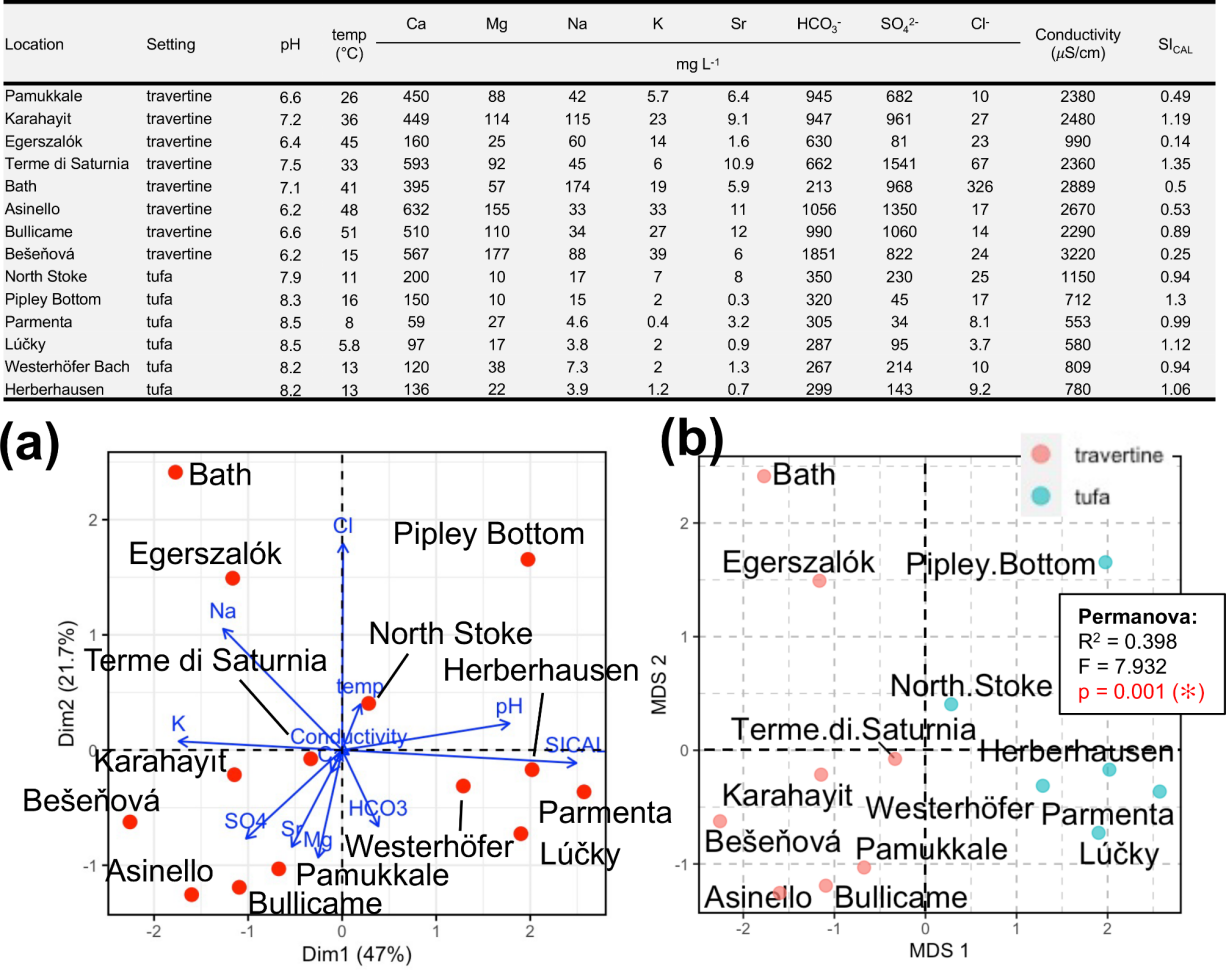
Geochemical data from all the studied travertine and tufa sites. Geochemical data listed in table are from references cited in Supplementary Table S1 and this study. (**a**) Principal Component Analysis biplot of geochemical data from travertine and tufa sites. The red dots mark 168 water analyses, which belong to 14 samples (8 travertines and 6 tufa); (**b**) Multidimensional Scaling Analysis of geochemical data. Statistical significance (*) was estimated based on Permanova test (*adonis2*, 999 permutations).

All biofilm samples, which were attached to the mineral growing surface of tufa and travertine, were collected in the spring-summer of 2021 and 2022 (Supplemental Fig. S1 and S2). The temperature of the water precipitating the travertine and tufa studied at the time of collection varied from 15 °C (Bešeňová) to 51 °C (Bullicame) and from 5.8 °C (Lúčky) to 16 °C (Pipley Bottom), respectively, with pH varying between 6.2 (Asinello, Bešeňová) and 7.5 (Terme di Saturnia) and between 7.9 (North Stoke) and 8.5 (Lúčky, Parmenta), respectively (Fig. 1). The chemical composition of the waters analysed from each sampling site chiefly represents a Ca-HCO_3_ saturated system (saturation index, SI_CAL_ = -0.8 to 1.3) with various admixtures of other major cations and anions depending on the source of the waters (Fig. 1). Principal component analysis (PCA) confirms geochemical composition similarities at travertine and tufa sites although the Bath water sample clearly differs from all samples (Fig. 1a), in respect to highest Na and Cl, and lowest HCO_3_ of all samples. Specifically, PCA shows that the tufa group is mostly dominated by higher pH, lower temperature, lower conductivity, lower HCO_3_, lower Cl and higher SI_CAL_ values, all of which are associated with positive loadings. By way of contrast, the travertine group is characterised by higher Sr, Mg, Ca and SO_4_ values associated with negative loadings and higher Na and K associated with positive loadings. Dim1 accounts for 47% of the total variance between the 12 components. This means that 53% of the variance is discarded, including the 21.7% that is associated with Dim2. These two geochemically different groups are also confirmed by multidimensional scaling analysis (MDS) in 2-dimensional space (MDS1, MDS2), although the Bath and Egerszalók travertines, as well as the North Stoke and Pipley Bottom tufas, are significantly distanced from other sampling sites (Fig. 1b). Permutational multivariate analysis of variance (Permanova) was computed to check whether differences between each group are significant. The calculated Permanova parameters (R^2^ < 0.4, F ~ 8, *p* < 0.001) show that the data obtained do fit the regression model and differences in water composition of travertine and tufa sites are statistically significant.

### Viral communities

**Fig. 2:**
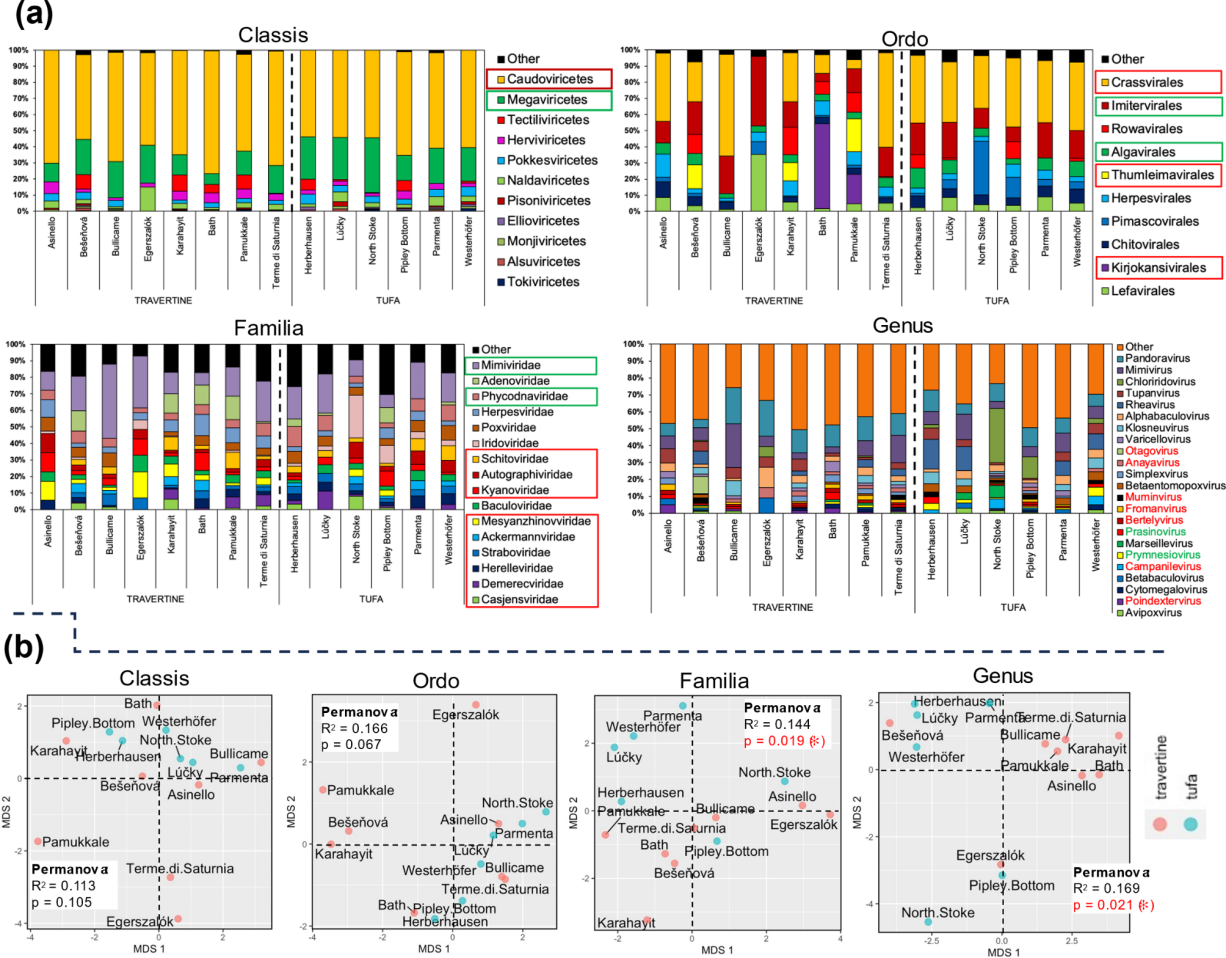
Viral compositions. (**a**) Data structures at different taxonomic levels for travertine and tufa biofilms; red and green rectangles – groups belonging to Caudoviricetes and Megaviricetes, respectively. (**b**) Multidimensional Scaling Analyses for different taxonomic levels. Statistical significance (*) was estimated based on Permanova test (*adonis2*, 999 permutations).

At the taxonomic level of *Classis*, two predominant groups are identified: Caudoviricetes (bacteriophages) and Megaviricetes (mega-sized viruses infecting algae and invertebrates). These groups were observed in both travertine and tufa biofilms, albeit in varying proportions (Fig. 2a). These groups constitute approximately 80% of all viruses identified in the analysed samples. The “other” group encompassed viruses for which the sum of contigs across all samples was less than 22 (cut-off level 22). At a lower taxonomic level (*Ordo*), a greater diversity of viruses becomes apparent. Notably, distinct groups emerge as the most abundant after excluding others (“other”) at the 40 contig cut-off level. The major groups include Crassvirales (bacteriophages), Imitervirales (mega-sized viruses, primarily icosahedral), Algalvirales (mega-sized viruses infecting, e.g., *Chlorella*-like algae), Thumleimavirales (tailed, icosahedral bacteriophages), Kirjokansvirales (tailed, icosahedral bacteriophages), and Lefavirales (infecting Arthropoda). At the *Ordo* taxonomic level, notable gaps in taxonomic positions are most pronounced. For instance, in the Egerszalók sample, the two main groups are Imitervirales and Lefavirales, whereas there is an absence of viruses belonging to the Caudoviricetes, even though, as previously demonstrated, this group is most abundantly represented at a higher taxonomic level. At the *Familia* taxonomic level, a considerable diversity of virus groups is evident (cut-off level for the sum of contigs set at 96). The significant groups belong to Mimiviridae and Phycodnaviridae (both groups containing algal mega-viruses, mostly icosahedral). Bacteriophages at *Familia* level are well-represented, such as the icosahedral Autographviridae and Kyanoviridae, infecting, among others, *Synechococcus* spp., Ackermannviridae (infecting Gammaproteobacteria) and Mesyanzhinovviridae, tailed, icosahedral phages with an indeterminate position at the *Ordo* level, are also numerous. At the *Genus* taxonomic level, numerous virus groups are identified, hence a cut-off level of 27 is adopted for data presentation. Key bacteriophages and algal viruses include *Otagovirus* (phage), *Anayavirus* (phage, host: *Mycobacterium*), *Muminvirus* (phage, host: *Flavobacterium*), *Fromanvirus* (phage, host: *Mycobacterium*), *Bertelyvirus* (phage, host: *Caulobacter*), *Prasinovirus* (phytoplankton host: Prasinophyceae), *Prymnesiovirus* (algal, host: *Chrysochromulina*), *Campanilevirus* (phage), and *Poindextervirus* (phage, host: *Caulobacter*). In all samples, the presence of two highly represented genera of some of the largest known viruses is noted: the non-icosahedral *Pandoravirus* and the icosahedral *Mimivirus* (host for both: *Acanthamoeba*).

The structure of a viral community composition does not allow for an ad hoc determination of whether the biofilms in travertine versus tufa samples differ from each other and at which taxonomic level. Therefore, these data were subjected to MDS analysis with the Permanova test to assess statistical significance (Fig. 2b). The results indicate that at the *Classis* and *Ordo* taxonomic levels, travertine and tufa samples do not group significantly differently. However, it is noteworthy that some travertine samples, over the analysed distances in the MDS analysis, tend to be somewhat separated from the other samples. In contrast, tufa samples appear to form a more cohesive group than travertine samples. At lower taxonomic levels (*Familia* and *Genus*), it can be demonstrated that travertine and tufa samples group separately (with significance levels of *p* = 0.019 and *p* = 0.021, respectively). It is worth noting the Egerszalók sample, regardless of the adopted taxonomic level, remains somewhat separated from other samples.

### Comparative analyses

**Fig. 3:**
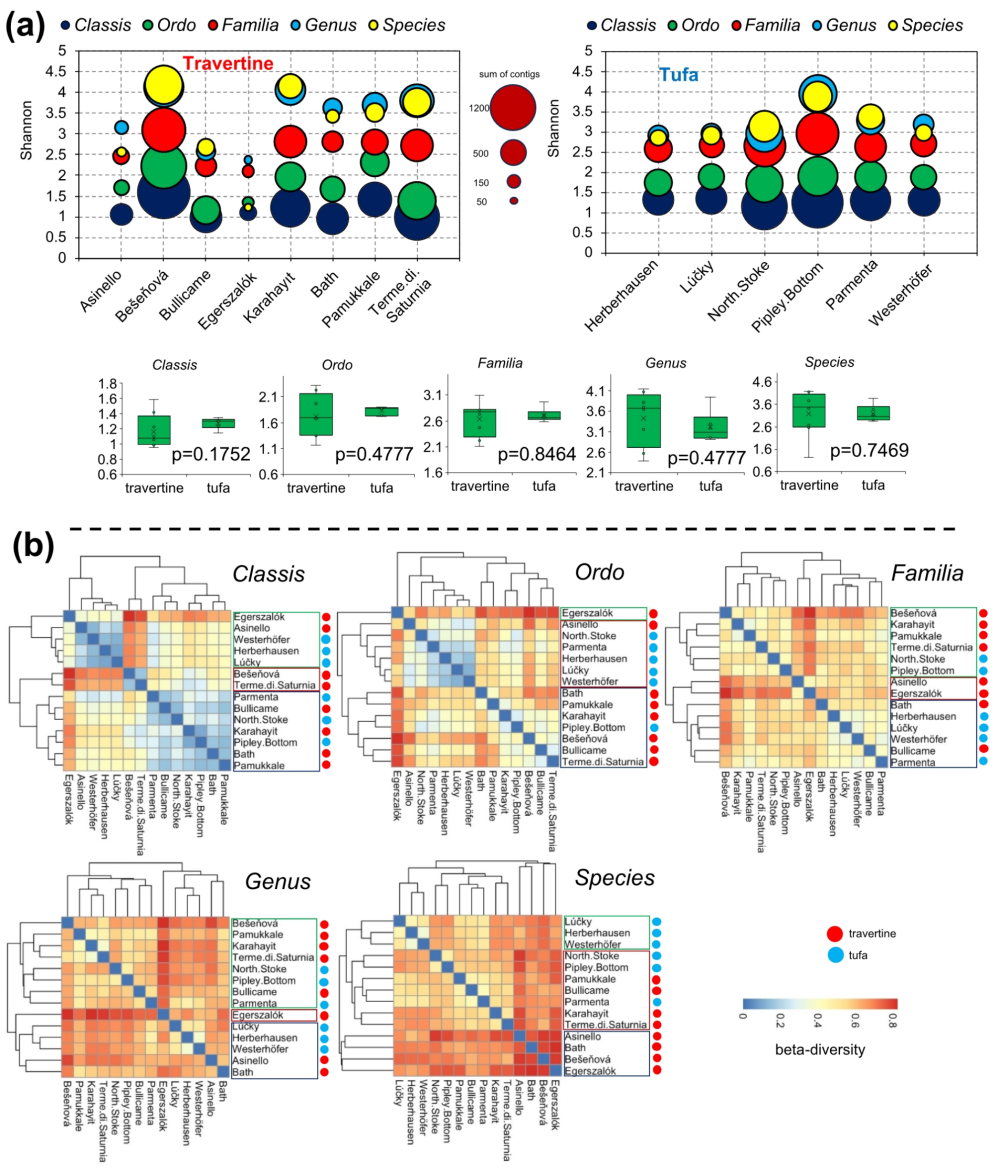
Comparative analyses. (**a**) Alpha-diversity (Shannon-Wiener index and sum of contigs), boxplots present: min, max, mean, median, first and third quartile. Statistical significance (p-values) based on the U-M-W test is added; (**b**) Hierarchical clustering based on Bray-Curtis dissimilarity matrices for different taxonomic levels.

Comparative analysis was used to elucidate the distinguishing characteristics of travertine and tufa with respect to their viral compositions. Alpha-diversity analysis reveals no significant differences within samples when comparing travertine and tufa *en bloc* (Fig. 3a, boxplots, Mann-Whitney test). The samples exhibit similar diversities (0.95–1.58, 1.16–2.31, 2.10–3.10, 2.37–4.14, 1.22–4.19; respectively for *Classis*, *Ordo*, *Familia*, *Genus* and *Species*), although some “outlier” samples are identified, such as Egerszalók, Asinello and Bullicame, likely because these are high-temperature (45–51 °C) springs, at the *Species* level. Additionally, travertine samples display a greater data spread compared to tufa. However, it is important to note that the samples vary in the number of contigs within fairly broad ranges, and it cannot be predetermined to what extent the observed diversity reflects actual biological diversity. Once again, the issue of “gaps” in virus taxonomy is evident, as the sum of contigs decreases with decreasing taxonomic levels in practically all samples.

Significant differences are evident in the beta-diversity analysis (Fig. 3b). The results show that as the taxonomic levels deepen, the diversity between samples increases. At the *Classis* level, three groups are distinguished, with samples exhibiting relatively low diversity between themselves. At a lower level (*Ordo*), only one group with samples of low diversity between themselves could be identified, and importantly, these are mostly tufa samples. At lower taxonomic levels, diversity between samples increases, especially within travertine samples. Cluster analysis reveals the existence of typically three main groups of samples, where either travertine or tufa dominate. However, it is worth noting that especially at lower taxonomic levels (*Familia to Species*), lower subgroups are formed by samples from travertine or tufa separately. Mixed subgroups are much less common.

### Identification of specific viral composition within travertine and tufa

**Fig. 4:**
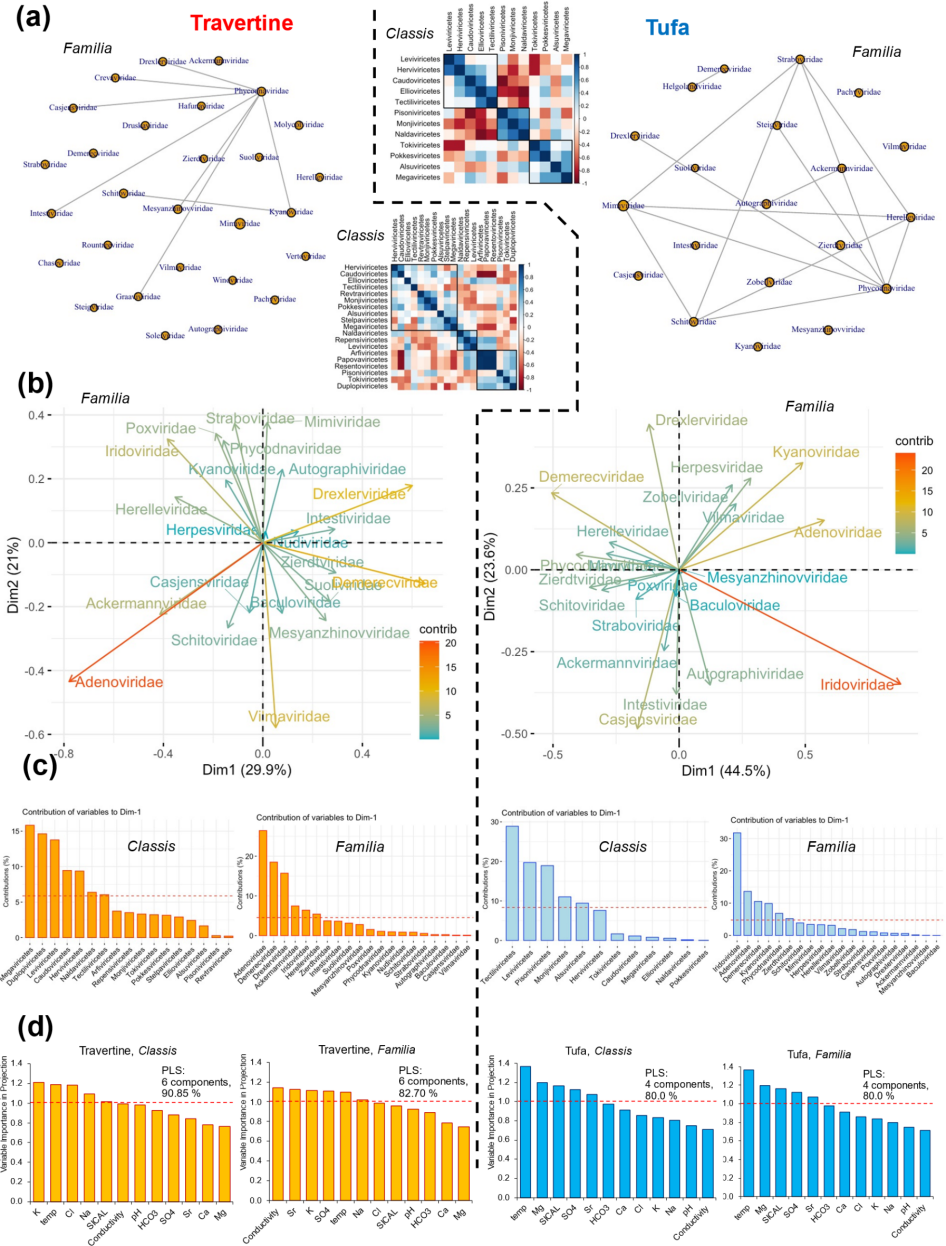
Specific viral compositions of travertine (left) and tufa (right) samples. (**a**) Correlogram showing significant Spearman correlations (α = 0.05) between viral groups (*Classis*) in travertine and tufa samples. Viral co-occurrence map (at *Familia* level) obtained from *SpiecEasi*; (**b**) PCA biplot of travertine and tufa viral composition at *Familia* level; (**c**) Contribution of variables (groups of viruses at *Classis* and *Familia* levels) to the first Principal Component. A reference dashed line is shown. Column with a contribution above the reference line can be considered as important in contributing to the dimension (a function included in the *FactoMineR* package); (**d**) PLS. Importance of geochemical variables (independent variables) in explaining the sum of squares for the dependent variable (position of individual observations in the viral data space based on PCA). A dashed line is marked at the conventional level VIP = 1, above which the influence of variables can be considered significant. The number of components and the percentage of explanation of the sum of squares for the dependent variables are given.

If it is assumed that the viral community may differentiate travertine and tufa samples, one would expect different co-occurrence patterns between virus groups. Such relationships are most easily visualised on a correlogram (Fig. 4a). The analysis reveals three clusters of viruses whose occurrences are somewhat correlated. These clusters include different virus groups depending on whether the samples are from travertine or tufa. For example, in tufa samples, the classes Pisoniviricetes, Monjiviricetes and Naldaviricetes form a cluster, and their abundances are correlated with each other. Such a relationship is not found in travertine samples. This issue can be approached in a more sophisticated way, revealing relationships at a deeper taxonomic level involving specific virus *Familiae* in travertine and tufa samples (Fig. 4a). The graph pattern illustrating the connections between viruses seems to differentiate travertine samples from tufa samples. The analysis shows a different level of complexity in these relationships. For example, in travertine samples, associations are observed between Phycodnaviridae and many other *Familiae*, but these groups are not interconnected. On the other hand, in tufa samples, a co-occurrence is apparent between practically all linked groups. This pattern is quite distinct from travertine samples.

PCA identified those groups of viruses whose contribution is greatest in explaining the variation in travertine and tufa samples (Fig. 4b, c). The viral composition clearly differentiates these two groups of samples. At the *Familia* level, the Adenoviridae (infecting eukaryotes), the Demerecviridae (infecting prokaryotes) and the Drexleviridae together with the Ackermannviridae (both tailed bacteriophages) are shown to be the groups with the largest contribution to the first dimension of principal components in travertines. At the *Classis* level, the groups are Megaviricetes (infecting eukaryotes), Duplopiviricetes (infecting eukaryotes) and Leviviricetes (infecting prokaryotes). Bacteriophages from the Caudoviricetes group and viruses infecting eukaryotes from the Herviviricetes group are also significantly differentiated. The proportion of viruses was determined differently for tufa samples. At the *Familia* level, the Iridoviridae group (infecting eukaryotes) contributes most to the variation and the Adenoviridae, Demerecviridae and Kyanoviridae (cyanophages) group contributes slightly less. At the *Classis* level, these are mainly Tectiliviricetes (infecting eukaryotes and prokaryotes), Leviviricetes and Pisoniviricetes (RNA-viruses infecting eukaryotes). Thus, if travertine and tufa samples are differentiated by different groups of viruses, it would be interesting to know whether the geochemical composition of the environment influences this differentiation. PCA allows the determination of the positioning vectors of each sample over multiple viral variables at the *Classis* and *Familia* levels. If these components are taken as dependent variables, it can be shown that the chemical characteristics of the environment influenced the virus variability of the travertine and tufa samples (Fig. 4d). It is remarkable that in the case of tufa the importance of the chemical variables in explaining the variability of the samples at both taxonomic levels is the same (temperature, Mg^2+^, SI_cal_, SO_4_^2−^). In contrast, in the case of travertine, the validity of the chemical variables depends on the taxonomic level describing the variability of the samples. Although here too, it is possible to distinguish features that are important in both cases (temperature and K^+^).

### Virus-host prediction

**Fig. 5:**
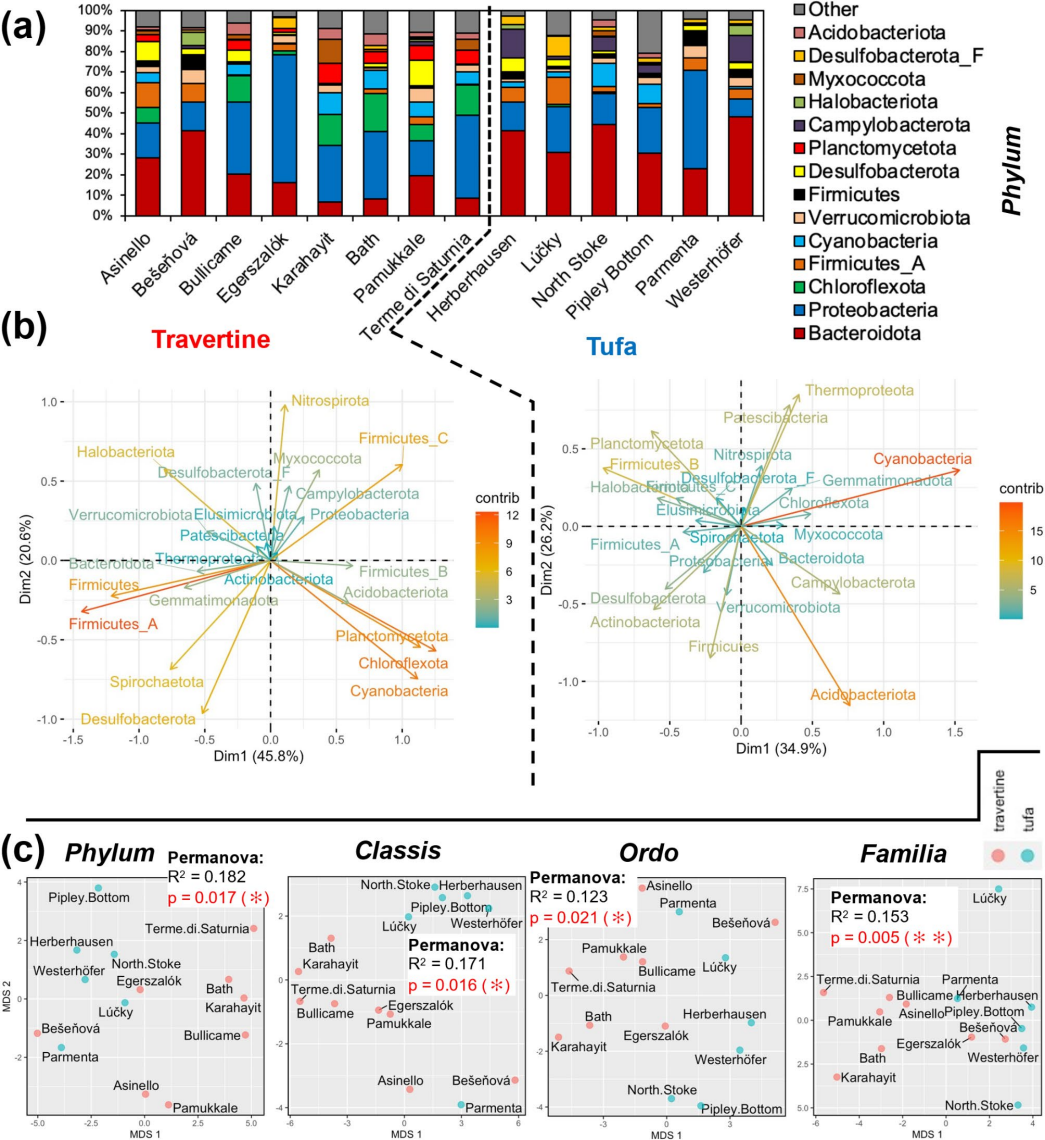
Virus-host prediction. (**a**) Data structure at the *Phylum* taxonomic level for travertine and tufa samples. (**b**) PCA biplot of travertine and tufa host compositions at the *Phylum* level. (**c**) Multidimensional Scaling Analyses for different taxonomic levels. Statistical significance (* *p* < 0.05, ** *p* < 0.001) was estimated based on Permanova test (*adonis2*, 999 permutations).

Prediction of microbial composition based on viral composition also shows that the two studied environments (tufa vs. travertine) are different (Fig. 5a). It should be noted that this analysis considered all phage sequences from which the presence of bacterial hosts can be predicted (always with some probability). Hence, not only viral sequences whose systematics are known were analysed, but also sequences that are known to belong to bacteriophages, although their taxonomic position is unclear. In this way, a more complete picture can be obtained to characterise and differentiate the carbonate environments studied. PCA shows that both environments are differentiated by separate microbial hosts (Fig. 5b). Firmicutes had the largest contribution to the variability of the travertine samples, with slightly smaller contributions from Cyanobacteria, Chloroflexota and Planctomycetota. In the tufa samples, the largest contribution came from Cyanobacteria and Acidobacteriota. Interestingly, statistically significant differentiation of the samples occurred at every taxonomic level tested (Fig. 5c). It appears that the travertine samples formed fewer compact groups in the MDS analysis compared to the tufa samples, although this strongly depended on the taxonomic level for which the analysis was performed.

## Discussion and conclusions

Multidimensional analysis (PCA, MDS) shows that travertine and tufa environments are geochemically differentiated with some exceptions (Bath, Egerszalók, North Stoke, Pipley Bottom). The main reason for such differences is likely to be the origin of the waters precipitating the two carbonate types. Namely, travertine is in general precipitated from thermal waters although the temperature factor is not the decisive one here (e.g., Bešeňová) but a deeper groundwater source could better characterise this type of freshwater carbonate, whereas tufa is associated with carbonates precipitated from freshwater at ambient temperature, mostly derived from rainfall permeating through soil and bedrock to the groundwater table and then emerging at springs. One additional feature of both terrestrial systems is a direct chemical precipitation process in association with microbial activities (i.e., the biofilms). Such microbially-mediated mineral precipitation is commonly dominated by cyanobacterial activity in these settings.

Metagenomic data reveal that the viral composition is related to the geochemical factors of the carbonate sedimentary environment. Numerous studies suggest that the distinct conditions governing the formation of travertines versus tufas likely influence the microbial community^37–39^. However, while this may seem obvious for bacteria and archaea, it is not always the same for viruses. First, the correlation between viral and bacterial compositions does not necessarily relate to the two carbonate-precipitating environments^40^. On the other hand, the link between viral and bacterial compositions may be closer in some soil environments although viral abundance can be negatively correlated with bacterial diversity^41^. Secondly, the abundance of viruses in the environment, their much smaller size and the virus-host relationships do not necessarily imply such obvious relationships as those found between bacteria and the environment. However, the results presented here show the specificity of viral composition for travertines and tufas. Hence, considering viral diversity, the environments of travertine and tufa formation strongly differ from each other regarding viral composition. It seems that travertines, due to their wider temperature range, may differ more from each other than tufa, as clearly shown by alpha- and beta-diversity analyses.

The viral variability of travertine and tufa samples is determined by evidently different groups of viruses. These are the groups that differentiate the samples from each other within travertines and tufa separately. Therefore, they should not be considered as specific viral proxies to distinguish the travertine and tufa environments. However, there are two important groups that may be relevant to the growth environments of biofilms and microbial mats: Caudoviricetes (infecting prokaryota) and Megaviricetes (infecting eukaryota including algae). Both groups are similarly abundant in tufa samples (not differentiating samples) but differently abundant in travertine samples (differentiating travertines). Evidently these environments create different conditions for the viruses (and their hosts) which is reflected in the viral variability. It should be noted that viral diversity could be diluted because different hosts, especially bacterial hosts, can be infected by many different viruses. Regardless, here the hypothesis that travertine and tufa environments really do differentiate viral composition has been tested, and it seems that this hypothesis has been confirmed. What is more, the ratio of Caudoviricetes to Megaviricetes (ratio of contig counts) can fairly well differentiate samples into the travertine or tufa group. Therefore, building on this, the Caudoviricetes/Megaviricetes ratio for travertine and tufa is expressed as follows (Supplemental Fig. S3): 4.84, 4.09 and 57% for travertines and 2.58, 2.39 and 52% for tufa (mean, median, and coefficient of variability, respectively). According to the non-parametric U-M-W test the Caudoviricetes/Megaviricetes ratios for travertine and tufa are substantially (but not statistically) different (*p* = 0.0612); however, more samples should be tested in the future.

This distinction between travertine-tufa samples is based on the fact that Caudoviricetes can be considered the most abundant group of viruses in the natural environment capable of infecting probably all known bacterial lineages^42–45^, and Megaviricetes the group infecting algae^46^, with both important in carbonate precipitation processes. Theoretically, the higher this ratio, the greater the share of microorganisms conducting heterotrophic processes in relation to photosynthetic processes driven by cyanobacteria, algae and other phototrophs. It has been demonstrated that viral capsids can be mineralised^12,23,47^ and if this fossilisation process could preserve genetic material then that may well carry important information about the composition of the microbial communities involved in the formation of older carbonate deposits. Currently, these are theoretical assumptions, but in the context of developing research on ancient DNA (aDNA; e.g.^48,49^), they are fully justified. The above assumptions can also be supported by virus-host analyses. Travertines and tufa are characterised by different host compositions as well as different proportions of bacterial groups and photosynthesising microorganisms. The host prediction analysis admittedly did not include eukaryotic organisms, but it can be assumed that the algal composition should also differentiate these two environments. It should be emphasised that our approach is novel because the exploration of characteristic viral proxies has usually concerned either strictly ecological studies or environments of possible sanitary-epidemiological significance^50–52^. It has been pointed out, however, that viral proxies can be an important element in the characterisation of various environments, including their relation to prokaryotic organisms and the resulting virus-host relationships^53^. Thus, from this point of view, the hypothesis was constructed that modern carbonate sedimentary environments are characterised by specific viral compositions. Importantly, many of the tufa and travertine streams studied herein have been monitored for years and the waters precipitating tufa and travertine are not so much affected by geographic location but are more fully dependent on the water source. Since all biofilm samples have been collected in spring-summer times, the proposed virus proxy is relevant for these modern carbonate settings, times when biofilms are more abundant than in the winter. In addition, oxygen content which would affect the microbial community in the analysed freshwater ecosystems probably is not varying much, contrasting with a microbial mat of a marine-lacustrine shoreline-tidal flat (e.g., Qatar^29^) where mats may be much thicker (> several cm). Therefore, in such thick microbial mats bacterial communities will vary with depth and with changing oxygen content downwards from the surface, as reported by e.g^29,54–56^. For the purpose of this study thin (2–5 mm thick, Supplementary Table S1 online) bulk biofilm samples have been analysed and oxygen stratification or influence will certainly have no effect on the proposed viral proxies.

The PLS analysis shows another important issue. Ideally, it should be the case that environmental chemical factors affect the viral variability of samples regardless of taxonomic level. This is the case in the tufa samples. This shows that tufa samples can be considered as a well-defined environment where chemical factors determine, or simply affect viral variability (note also: alpha-diversity at different taxonomic levels is similar for samples; low variability of this parameter within taxonomic levels exists). However, for travertine samples, alpha-diversity is more variable and, in addition, in some samples strongly decreases with the depth of the taxonomic levels. This can also be seen in the beta-diversity at the *Species* level, where travertine samples are more variable among themselves. Therefore, here the impact of chemical factors may be less clear, and this can be seen in the differential importance of chemical factors in explaining virus variation at the *Classis* and *Familia* taxonomic levels (see Fig. 4d for travertines).

The question is whether human influence can disturb the results of the viral composition, which groups the samples into travertines and tufa. This influence probably manifests itself in a greater discrepancy in the data for the travertine samples because some of them were exposed to more anthropopression, in contrast to the tufa sites. On the other hand, all the studied sites are subject to anthropopression due to being surrounded by agriculturally used fields. Pamukkale, Terme di Saturnia, Karahayıt, Bath are tourist destinations and there the impact may be greatest. Samples from these sites, however, do not group separately from other travertines. Only Bešeňová appears to be the most heavily subjected to anthropopression and here the possible human influence on the composition of the viral communities may be highest. In most analyses presented here, the site generally outliers the other travertines. But even in this case the effect is not always obvious e.g. the MDS analysis at the *Genus* level shows a clustering of the Bešeňová sample together with the sites most likely to be affected by the weakest anthropopression (Herberhausen and Westerhöfer). It is likely that the strong contribution of Adenoviridae to the variability of these samples could have been the result of human influence. Similarly, the differential influence of Caudoviricetes on the variability of the travertine and tufa samples could be considered, since, if human influence was indeed present, then the influx of other bacterial flora into the environment (Caudoviricetes are bacteriophages) could also have been marked. This aspect is interesting and important, as it is possible that based on a viral analysis such an influence can be recognised. Egerszalók has formed from water which discharges from an artificial well. The site is quite young and in the last decades the waterflow on the travertine hill has been controlled by human activity; however, the travertine hill is protected from tourists^57^. This is clearly different from the situation at Bešeňová^58^. This site is located within meadows used for ages by domestic animals; tourist activity is also very high there.

In conclusion, the proposed viral proxy chiefly based on the environmental and anthropogenic factors gives a new opportunity to study similar microbially-rich sedimentary systems in which microorganisms form biofilms as important mineral-forming agents. This new finding opens up the potential to use viruses as another tool to differentiate modern and active sedimentary environments which can lead to a better understanding of changes occurring in freshwater ecosystems in which mineral precipitation takes place. This new approach can also be applied and tested in marine systems with similar mineral-microbial characteristics.

## Methods

### Sample collection

Biofilms (2–5 mm thick, ~ 3–5 cm^2^ volume) attached to the travertine surfaces of deposition have been collected at Asinello (Italy), Bath (United Kingdom), Bešeňová (Slovakia), Bullicame (Italy), Egerszalók (Hungary), Karahayıt ‘Kızılsu’ (Turkey), Pamukkale (Turkey), and Terme di Saturnia (Italy); sites where biofilms associated with freshwater tufa surfaces of deposition have been collected are Lúčky (Slovakia), North Stoke and Pipley Bottom (United Kingdom), Parmenta (Italy), Herberhausen and Westerhöfer (Germany) (Supplemental Table S1). Biofilm samples were placed in sterile 15 mL Falcon tubes and filled with their original water. Biofilms growing on the tufa-precipitating mineral substrate are green-brown whereas those developing on travertine have various colours from green to brown to red, suggesting more varied microbial communities in the latter than in the former. Temperature, pH and conductivity were measured directly at each site while collecting samples.

### Geochemical analysis of water

pH, temperature and chemical composition of water from the studied sampling sites have been obtained from published data^57,59–69^, except Bath, Bešeňová, Lúčky, and Pipley Bottom. Saturation index of calcite (SI_CAL_) was calculated using the *phreeqc* database in PHREEQC v. 3.7.3 ^70^. pH, conductivity and temperature were measured in the field using a Hach HQ40d Multi-Parameter Meter with PHC101 pH electrode (± 0.02 pH, ± 0.3 °C) and CDC401 conductivity cell (± 0.5%, ± 0.3 °C) IntelliCAL probes. Alkalinity was measured by titration against 0.005 M HCl. Anions were analysed by ion chromatography (IC; Dionex ICS-6000) and cations by inductively coupled plasma optical emission spectroscopy (ICP-OES; Agilent 710 ICP-OES).

### Viral communities

The genomic DNA from the environmental samples was isolated using an EURx kit for complex matrix (Soil DNA Purification Kit, no E3570, EURX Ltd. Poland) according to the protocol of the manufacturer. The protocol assumes the mechanical homogenisation of the samples to release the cells from the rock matrix. The samples used for isolation were moist solid material with associated biofilm. No water was taken for isolation along with the solid sample except that associated with the biofilm and sample moisture. The isolated genomic DNA was subjected to metagenomic analysis in Genomed S.A. (Warsaw, Poland). DNA samples were mechanically fragmented with Focused-ultrasonicator Covaris E220. Libraries were prepared with NEBNext® Ultra™ II DNA Library Prep Kit for Illumina® (New England Biolabs, E7645L) according to manufacturer protocol. Sequencing was performed on an Illumina NovaSeq 6000 system with paired end technology, 2 × 150nt. A 2x greater read depth (80 million pairs instead of the standard 40 million pairs) was used to facilitate detection of viral reads. All the bioinformatic computations were carried out on a high-performance computing infrastructure PLGrid (HPC Centers: ACK Cyfronet, AGH University of Krakow). Raw fastq files were pre-processed with fastp (0.23.4)^71,72^ in order to enhance the quality of raw reads. Then, a *de novo* contig assembly was performed with megahit (1.2.9)^73^ using *--presets meta-large*. Contigs were used for (i) taxonomic profiling and (ii) virus-host prediction.

### Taxonomic profiling

Contigs were subjected to taxonomic assignment. A k-mer based kraken2 software (2.1.3; standard database, release date: 09.10.2023)^74,75^ was used to obtain classification reports. Then, bracken (2.9)^76^ was applied on reports to estimate the relative abundances of species.

### Virus-host prediction

Virsorter (2.2.4)^77^ was used to separate viral contigs. Then, exported viral contigs were additionally analysed with checkV (1.0.1)^78^ to ensure the viral sequences were devoid of any contaminants, such as host sequences. Eventually, cleaned viral contigs were analysed with iphop (1.3.1; database release: August 2023)^79–83^ to get the overview of hosts associated with detected viral sequences.

### Comparative analyses and statistics

Multidimensional analyses, correlograms, diversity tests within and between samples were computed using R^84^. If the analyses required the use of zero imputation and normalization due to compositional data, tools from the *compositions* and *zCompositions* packages (centered log-ratio *clr*, and Bayesian-Multiplicative replacement of count zeros *cmultRepl*) were used^85–87^. Principal Component Analysis was conducted using *PCA* from package *FactoMineR*, visualized with package *factoextra*. Classical Multidimensional Scaling (MDS) was computed with *cmdscale* from package *MASS*, visualized with *ggplot2*^88^. Permutational Multivariate Analysis of Variance Using Distance Matrices^89^ was performed with *adonis2* from package *vegan* (permutations = 999, method = “bray”). Correlograms were performed with *cor* from package *corrplot* (method = “spearman”, visualised with corrplot, hclust.method = “ward.D”, addrect = 3). Alpha-diversity^90^ was computed with *diversity* from package *vegan* (index = “shannon”). Beta-diversity^85^ was computed with *vegdist* from package *vegan* (method = “bray”, visualised with *pheatmap* from package *pheatmap*). The statistical analyses including nonparametric test for differences (U-Mann-Whitney test; U-M-W test) were conducted with Statistica 13 software (StatSoft Inc., Tulsa, OK, USA).

### Identification of specific viral composition

First, association network analysis was performed using *SpiecEasi* – Sparse InversE Covariance estimation for Ecological Association and Statistical Inference^91^. To identify the viral composition specific to travertine or tufa samples, the following algorithm was implemented. Taxonomic levels *Classis* and *Familia* were selected. The *Ordo* level contains many lacks in taxonomy, while lower levels often remain undefined for individual contigs. Subsequently, a cut-off level was adopted to reduce the number of contigs incidentally occurring in individual samples (a high number of single reads strongly affects the PCA analysis). Cut-off levels of 5 were adopted for the *Classis* level and 20 for the *Familia* level (the sum of contigs from samples for a given taxon is < 5 and < 20, respectively). Next, the data was transformed by filling in zero values and performing transformation using Bayesian-Multiplicative Replacement of count zeros *cmultRepl* and centred log-ratio *clr* (R packages *zCompositions* and *compositions*). Principal Component Analysis was then performed to obtain contributions of individual virus groups to the first PCA dimension (R package *FactoMineR*, *pca(scale.unit = FALSE #data were already transformed)*, *fviz_contrib(choice = “var”*,* axes = 1*,* top = 20)*). Subsequently, coordinates of principal components for each observation (Dim.1, Dim.2, Dim.3, Dim.4, and Dim.5) were determined, defining the position of individual observations in the viral data space. These coordinates were then used as dependent variables in Partial Least Squares (PLS) analysis, employing geochemical data as independent variables. The nonlinear iterative partial least squares (NIPALS) algorithm was used in Statistica 13 software (StatSoft Inc., Tulsa, OK, USA). In this way, factors with the greatest contribution to explaining viral variability at the *Classis* and *Familia* levels within travertine and tufa samples were determined separately.

## Electronic supplementary material

Below is the link to the electronic supplementary material.

Supplementary Material 1

## Data Availability

The datasets generated during and/or analysed during the current study are available in the SRA repository, https://www.ncbi.nlm.nih.gov/sra/PRJNA957956.
