# Supplementary material for: Biofilms in modern CaCO3-supersaturated freshwater environments reveal viral proxies
Source: Sci Rep. 2024 Oct 29;14:25889. doi: 10.1038/s41598-024-75998-7 (PMC11519349; doi:10.1038/s41598-024-75998-7)
Supplement: Supplementary file 1 — Supplementary Material 1 [file 41598_2024_75998_MOESM1_ESM.docx]

**Biofilms in modern CaCO_3_-supersaturated freshwater environments reveal viral proxies**

Mirosław Słowakiewicz^a*^, Andrzej Borkowski^b^, Edoardo Perri^c^, Paweł Działak^b^, Ezher Tagliasacchi^d^, Michał Gradziński^e^, Sándor Kele^f,g^, Lars Reuning^h^, Tom Kibblewhite^i^, Fiona Whitaker^i^, R. Pamela Reid^j^, Maurice E. Tucker^i^

^a^Faculty of Geology, University of Warsaw, Warsaw, Poland

^b^Faculty of Geology, Geophysics and Environmental Protection, AGH University of Krakow, Kraków, Poland

^c^Dipartimento di Biologia Ecologia e Scienze della Terra, Università della Calabria, Rende, Italy

^d^Faculty of Engineering, Pamukkale University, Kınıklı Campus, Denizli, Turkey

^e^Institute of Geological Sciences, Jagiellonian University, Kraków, Poland

^f^HUN-REN Research Centre for Astronomy and Earth Sciences, Institute for Geological and Geochemical Research, Budapest, Hungary

^g^CSFK, MTA Centre of Excellence, Budapest, Hungary

^h^Institute of Geosciences, Kiel University, Kiel, Germany

^i^School of Earth Sciences, University of Bristol, Bristol, UK

^j^Rosenstiel School of Marine, Atmospheric and Earth Science, University of Miami, Miami, USA

^1^To whom correspondence may be addressed. Email: m.slowakiewicz@gmail.com or m.slowakiewicz@uw.edu.pl.

Table S1. Location, coordinates, type and mineralogy of the studied samples. All biofilms are relatively thin (2 to 5 mm thick) and attached to the mineral surface. Mineralogy has been analysed by X-ray diffraction and selected area electron diffraction in the cited references. Mineral phases shown are the most predominant or solely forming the travertine or tufa.

| **Location** | **Country (Region)** | **Coordinates** | **Type of samples** | **Mineralogy of carbonate deposits** | **Sampling season** |
| --- | --- | --- | --- | --- | --- |
| Egerszalók travertine | Hungary (Egerszalók) | 47°51'11.95"N, 20°20'02.83"E | Green biofilm samples (2-3 mm thick) from distal part of system, collected below and farther from mound (20-25 m from well) | Calcite, aragonite ^1^ | March 2022 (spring) |
| Sacred Spring travertine (Bath) | UK (Bath) | 51°22'50.67"N, 2°21'34.60"W | Green-brown biofilm (3 mm thick) with precipitates close to spring (20 m) | Calcite, ferrihydrite ^2^ | July 2022 (summer) |
| Terme di Saturnia travertine | Italy (Grosetto, Tuscany) | 42°39'31.23"N, 11°30'59.61"E | Greenish and yellow biofilm (2 mm thick) on surface of travertine, distal part of spring (~1 km) and close to pools | Calcite ^3^ | May 2021 (spring) |
| Bullicame travertine | Italy (Viterbo, Lazio) | 42°25'12.98"N, 12°04'22.07"E | Green and yellowish biofilm (3-4 mm thick) within travertine, close to hot spring (~ 30 m) | Calcite, aragonite ^4,3^ | May 2021 (spring) |
| Asinello travertine | Italy (Viterbo, Lazio) | 42°23'57.01"N, 12° 3'31.71"E | Green, yellowish and beige biofilm (5 mm thick) with travertine close to spring (2-3 m) | Calcite, probable aragonite (no data) ^2^ | March 2022 (spring) |
| Karahayıt ‘Kızılsu’ travertine | Turkey (Denizli) | 37°58'2.42"N, 29°6'9.30"E | Yellowish, green and brownish biofilm (3-4 mm thick) on surface of precipitates, close to hot spring (30 m) | Calcite, aragonite ^5^ | August 2021 (summer) |
| Bešeňová travertine | Slovakia (Bešeňová) | 49°06'13.83"N, 19°26'09.95"E | Reddish-brown biofilm (5 mm thick) attached to travertine, 4 m from spring, in channel over travertine cascade | Calcite, goethite ^2^ | April 2022 (spring) |
| Pamukkale travertine | Turkey (Denizli) | 37°55'24.46"N, 29°07'23.28"E | Brown, yellow and green biofilm (2-3 mm thick) on surface of precipitates and interlayered with travertine, samples collected from channel farther from spring (~400-450 m) | Calcite, aragonite ^5^ | August 2021 (summer) |
| North Stoke tufa | UK (Bath) | 51°25'12.52"N, 2°25'40.84"W | Green-brown biofilm (3 mm thick) with tufa from cascade (90 m from spring) | Calcite | June 2022 (spring) |
| Pipley Bottom tufa | UK (Bristol) | 51°25'26"N, 2°25'52"W | Green-brown biofilm (3 mm thick) with tufa in stream (150 m from spring) | Calcite | June 2022 (spring) |
| Parmenta tufa | Italy (Buonvicino, Cosenza) | 39°41'05''N  15°55'03''E | Green-brown biofilm (3 mm thick) with tufa in stream (no spring, just a short stream in a 1-km long valley) | Calcite ^6,7^ | April 2022 (spring) |
| Lúčky tufa | Slovakia (Lúčky) | 49°07'47.40"N, 19°24'11.39"E | Green and brownish-green biofilm (5 mm thick) with tufa in stream (~300-500 m from spring) | Calcite ^8^ | April 2022 (spring) |
| Westerhöfer tufa | Germany (Kalefeld) | 51°45'44.0"N 10°05'35.0"E | Green biofilm (5 mm thick) with tufa in stream (90 m from the spring) | Calcite ^9^ | April 2022 (spring) |
| Herberhausen tufa | Germany (Gőttingen) | 51°32'20.4"N 9°59'26.3"E | Brown biofilm (3-4 mm thick) with tufa from cascade (100 m from the spring) | Calcite ^10^ | April 2022 (spring) |


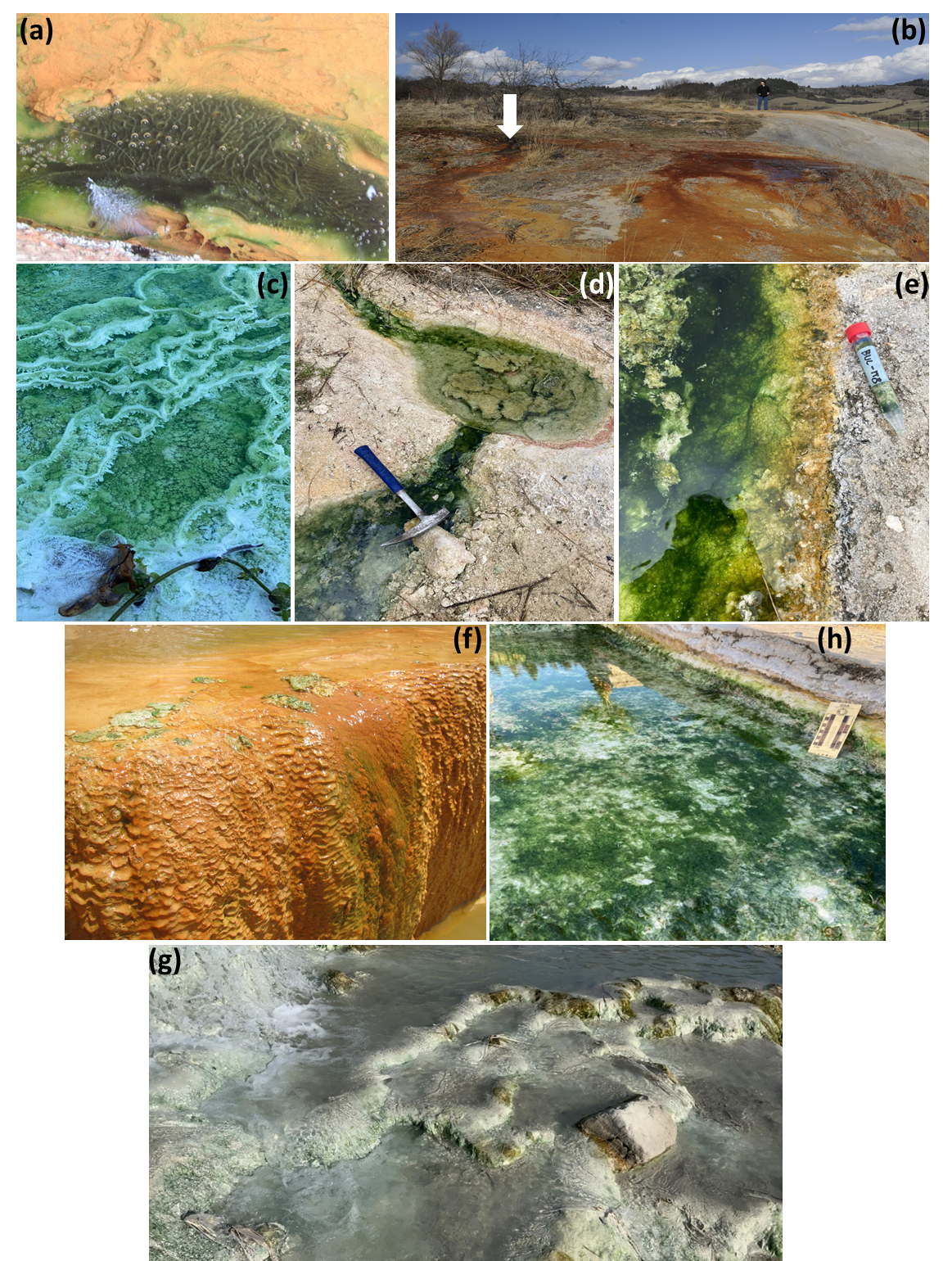


Fig. S1. Images of the studied biofilm from travertine samples at the mesoscale. (a) Green rippled and smooth biofilm in very shallow, fast-flowing channel from Sacred Spring to Great Bath, Bath, within which calcite and ferrihydrite being precipitated. Field of view 40 cm across; (b) Sampling site (arrow) at Bešeňová, top of the travertine cascade to the right. Biofilms with brownish colour. Person for scale 177 cm tall; (c) Green biofilm collected at the distal parts of the Egerszalók travertine mound, below the cascades and approximately 25-30 m away from the thermal well. Falcon tubes in this and other images are 12 cm long; (d) Circular vent orifice and proximal channel of travertine at Asinello with dark green biofilm. Hammer 28 cm long; (e) Proximal channel of the Bullicame travertine system. Channel floor is covered with green biofilm. Tube is 15 cm long; (f) Reddish to brown travertine covered with green biofilm at Karahayıt “Kızılsu (red water)”. Field of view 40 cm across; (g) Proximal channel of Pamukkale travertine. Channel floor is covered with green biofilm. Scale is 10 cm; (h) Distal part of the terraced slope of Terme di Saturnia travertine where travertine pool rims are partly covered with green biofilm. Field of view 2 m across.


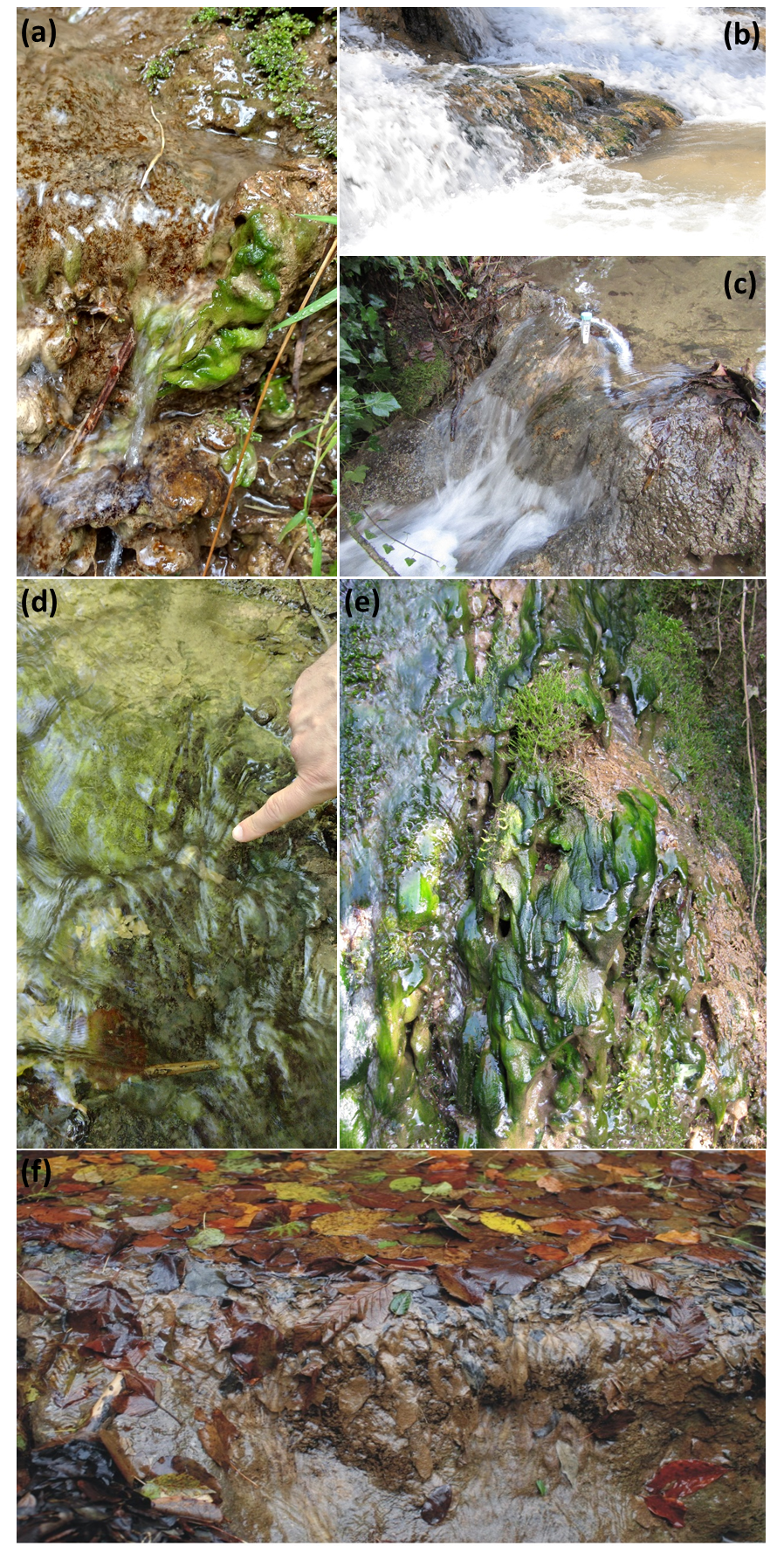


Fig. S2. Images of the studied biofilm from tufa samples at the mesoscale. (a) Brownish biofilms from the top of a small tufa cascade in Herberhausen. Green patch in the middle right of the picture is the green algae-dominated biofilm. Field of view ~5 cm across; (b) Upper part of the tufa depositing system at Lúčky; small cascade ca. 0.5 m high covered with green biofilm composed mostly of *Vaucheria* sp., *Phormidium* sp. and diatoms; (c) Barrage, one metre across, with dark green biofilm beneath fast-flowing water, Pipley Bottom, Bath; (d) Cyanobacteria dominated green biofilm on tufa stromatolite in the middle of a karst-water stream (Westerhöfer). Field of view ~20 cm across; (e) Cascade tufa with moss and green filamentous microbial biofilm, field of view 50 cm across, North Stoke, Bath; (f) Barrage on the Parmenta stream (Calabria) with leaves above and biofilm over steep slope. Field of view 50 cm.


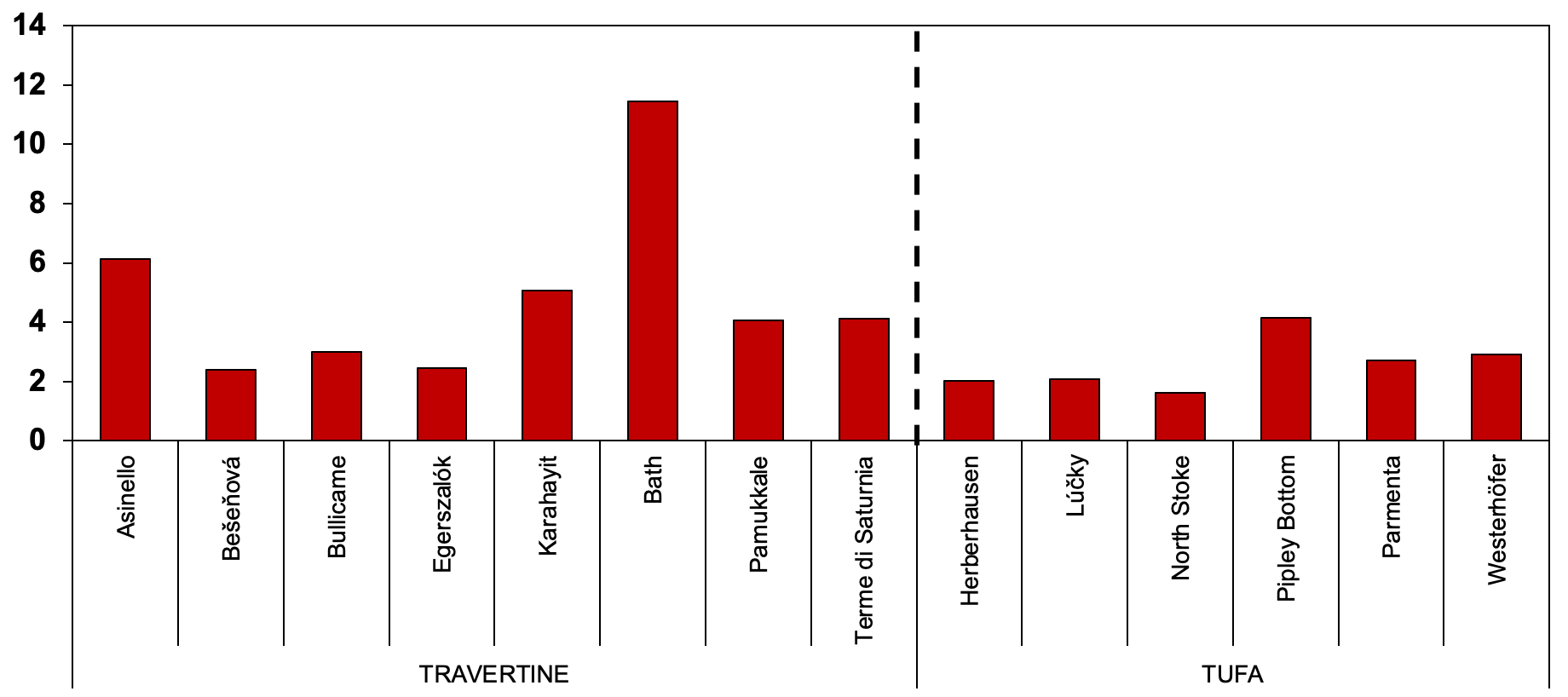


Fig. S3. The Caudoviricetes / Megaviricetes ratio based on viral compositions of travertine and tufa samples.

**References**

1. Kele, S. *et al.* Chemical and stable isotope composition of recent hot-water travertines and associated thermal waters, from Egerszalók, Hungary: Depositional facies and non-equilibrium fractionation. *Sedimentary Geology* **211**, 53–72 (2008).

2. Słowakiewicz, M. *et al.* Viruses participate in the organomineralization of travertines. *Scientific Reports* **13**, 11663 (2023).

3. Della Porta, G., Hoppert, M., Hallmann, C., Schneider, D. & Reitner, J. The influence of microbial mats on travertine precipitation in active hydrothermal systems (Central Italy). *The Depositional Record* **8**, 165–209 (2022).

4. Di Benedetto, F. *et al.* Biotic and inorganic control on travertine deposition at Bullicame 3 spring (Viterbo, Italy): A multidisciplinary approach. *Geochimica et Cosmochimica Acta* **75**, 4441–4455 (2011).

5. Özkul, M. *et al.* Comparison of the Quaternary travertine sites in the Denizli extensional basin based on their depositional and geochemical data. *Sedimentary Geology* **294**, 179–204 (2013).

6. Manzo, E., Perri, E. & Tucker, M. E. Carbonate deposition in a fluvial tufa system: processes and products (Corvino Valley – southern Italy). *Sedimentology* **59**, 553–577 (2012).

7. Perri, E., Słowakiewicz, M., Perrotta, I. D. & Tucker, M. E. Biomineralization processes in modern calcareous tufa: Possible roles of viruses, vesicles and extracellular polymeric substances (Corvino Valley – Southern Italy). *Sedimentology* **69**, 399–422 (2022).

8. Gradziński, M. Factors controlling growth of modern tufa: results of a field experiment. *Geological Society, London, Special Publications* **336**, 143–191 (2010).

9. Schneider, D., Reimer, A., Hahlbrock, A., Arp, G. & Daniel, R. Metagenomic and metatranscriptomic analyses of bacterial communities derived from a calcifying karst water creek biofilm and tufa. *Geomicrobiology Journal* **32**, 316–331 (2015).

10. Arp, G. & Reimer, A. Hydrochemistry, biofilms and tufa formation in the karstwater stream Lutter (Herberhausen near Göttingen). *Göttingen Contributions to Geosciences* 77–82 (2014).
